# A spatial temporal analysis of the *Fusarium graminearum* transcriptome during symptomless and symptomatic wheat infection

**DOI:** 10.1111/mpp.12564

**Published:** 2017-08-08

**Authors:** Neil A. Brown, Jess Evans, Andrew Mead, Kim E. Hammond‐Kosack

**Affiliations:** ^1^ Department of Biointeractions and Crop Protection Rothamsted Research Harpenden, Hertfordshire AL5 2JQ, UK; ^2^ Computational and Analytical Sciences Rothamsted Research Harpenden, Hertfordshire AL5 2JQ, UK

**Keywords:** deoxynivalenol, fungal effectors, Fusarium head blight, secondary metabolism, symptomless disease, transcriptome, wheat

## Abstract

Fusarium head blight of wheat is one of the most serious and hazardous crop diseases worldwide. Here, a transcriptomic investigation of *Fusarium graminearum* reveals a new model for symptomless and symptomatic wheat infection. The predicted metabolic state and secretome of *F. graminearum* were distinct within symptomless and symptomatic wheat tissues. Transcripts for genes involved in the biosynthesis of the mycotoxin, deoxynivalenol, plus other characterized and putative secondary metabolite clusters increased in abundance in symptomless tissue. Transcripts encoding for genes of distinct groups of putative secreted effectors increased within either symptomless or symptomatic tissue. Numerous pathogenicity‐associated gene transcripts and transcripts representing PHI‐base mutations that impacted on virulence increased in symptomless tissue. In contrast, hydrolytic carbohydrate‐active enzyme (CAZyme) and lipase gene transcripts exhibited a different pattern of expression, resulting in elevated transcript abundance during the development of disease symptoms. Genome‐wide comparisons with existing datasets confirmed that, within the wheat floral tissue, at a single time point, different phases of infection co‐exist, which are spatially separated and reminiscent of both early and late infection. This study provides novel insights into the combined spatial temporal coordination of functionally characterized and hypothesized virulence strategies.

## Introduction

Fusarium head blight (FHB) of wheat, caused mainly by the ascomycete fungus *Fusarium graminearum*, is one of the most serious and hazardous crop diseases worldwide (http://scabusa.org/). The main consequence of FHB is that trichothecene mycotoxins, such as deoxynivalenol (DON), accumulate in the grain, presenting a food safety risk and health hazard to humans and animals (Goswami and Kistler, 2004). New approaches to control FHB are needed to improve wheat grain yield, quality and safety. The *F. graminearum*‐wheat infection process is unique when compared with other model and non‐model fungal pathosystems. Infection starts with or without the formation of complex infection structures, such as infection cushions, which penetrate wheat floral tissue (Boenisch and Schaefer, 2011). Within the wheat host plant, *F. graminearum* infections commence with extracellular hyphae advancing between live host cells without causing visible disease symptoms, reminiscent of an apoplastic biotroph. This prolonged period of symptomless infection extends for over a centimetre beyond visible disease symptoms. The subsequent development of disease symptoms behind the infection front is represented by the bleaching of the wheat tissue. This coincides with the death of the wheat cells that are surrounded by fungal hyphae and the intracellular colonization of these dead plant cells, similar to a necrotrophic pathogen (Brown *et al*., 2010, 2011). Therefore, the extremities along the continuum of the *F. graminearum*–wheat interaction are phenotypically distinct at the microscopic and macroscopic level. Henceforth, these two spatially and temporally separated infected plant tissues are described according to their macroscopic appearance and are referred to as symptomless or symptomatic.

*Fusarium graminearum* is known to secrete a sophisticated cocktail of enzymatic and non‐enzymatic proteinaceous virulence factors in combination with host‐ and non‐host‐specific, toxic and non‐toxic metabolites to cause disease. These include: (i) the sesquiterpernoid DON (Cuzick *et al*., 2008; Ehrlich and Daigle, 1987); (ii) the Fgl1 secreted lipase (Bluemke *et al*., 2014); (iii) the iron‐scavenging secreted siderophore triacetyl fusarinine C (TAFC) (Oide *et al*., 2006); and (iv) carbohydrate‐active enzymes (CAZymes) that can readily deconstruct plant cell walls and induce host cell death (Brown *et al*., 2010, 2011, 2012; Sella *et al*., 2013; Sperschneider *et al*., 2016; Zhang *et al*., 2012). Prior to the discovery of symptomless *F. graminearum* wheat infection, a species‐specific Affymetrix array was developed and utilized to interrogate the *F. graminearum* transcriptome under various *in vitro* and *in planta* conditions (Gueldener *et al*., 2006; Lysoe *et al*., 2011; Stephens *et al*., 2008). These studies documented the transcription profile of *F. graminearum* during barley head, wheat head and wheat crown (stem‐based) infection, plus wheat stems during the development of perithecia, in addition to providing an *in vitro* nutrient‐rich and nutrient‐poor comparison. However, these time‐dependent studies did not specifically isolate the distinct phases of fungal infection, and therefore obscured transcriptional modulations significant to the recently identified symptomless and symptomatic wheat head infection process. Later, laser‐capture micro‐dissection (LCM) and a single tissue‐type wheat coleoptile stem assay, which is distinct from most wheat head disease situations, were used to evaluate three similar phases of infection, namely: (i) covert penetration; (ii) rapid proliferation; and (iii) overt destruction (Zhang *et al*., 2012). The LCM study revealed the involvement of the glyoxylate cycle, fungal mechanisms to scavenge reactive oxygen species and the secretion of plant cell wall‐degrading CAZymes during infection.

In this study, a genome‐wide transcriptomic investigation is used to examine the early phases of the *F. graminearum*–wheat head interaction, exploring both symptomless and symptomatic infections. These analyses generate new spatially and temporally linked datasets, permitting the development of a descriptive model for the *F. graminearum* transcriptome along the continuum of wheat head infection. This study reveals the coordination of previously characterized and several putative virulence strategies potentially deployed within symptomless and symptomatic wheat tissues to facilitate infection. The availability of these spatial temporal datasets and the generation of a model for the early wheat infection process occurring as the advancing hyphae colonize the wheat rachis will facilitate the identification of additional genes involved in the infection process.

## Results

### The identification of *F. graminearum* infection in symptomless and symptomatic wheat rachis tissues

Conidia of *F. graminearum* were inoculated into the central spikelets of the wheat head at anthesis. During the first 5 days of *F. graminearum* infection, disease symptoms were confined to the inoculated spikelet. After 7 days, symptoms began to radiate out from the inoculated spikelet and spread along the rachis, whereas the non‐inoculated spikelets remained symptomless. After 9 and 12 days, the majority of the spikelets and rachis internodes of the wheat head presented disease symptoms (Fig. S1, see Supporting Information). The distinct symptomless and symptomatic phases of the *F. graminearum* infection process were isolated by sequentially harvesting rachis internodes below the inoculated spikelet throughout the course of wheat infection (Fig. S1). The macroscopic and subcellular characteristics of infection within these rachis tissues have been described previously in an extensive histological study (Brown *et al*., 2010). This showed that *F. graminearum* infection of wheat rachis tissue commenced with the intercellular colonization of the apoplast, without the appearance of macroscopic or microscopic disease symptoms, and hence was termed ‘symptomless infection’. Subsequently, disease symptoms developed as a result of the induction of host cell death and the intracellular penetration of dead plant cells, which was accompanied by the proliferation of fungal biomass. This later phase was therefore termed ‘symptomatic infection’ (Brown *et al*., 2010).

**Figure 1:**
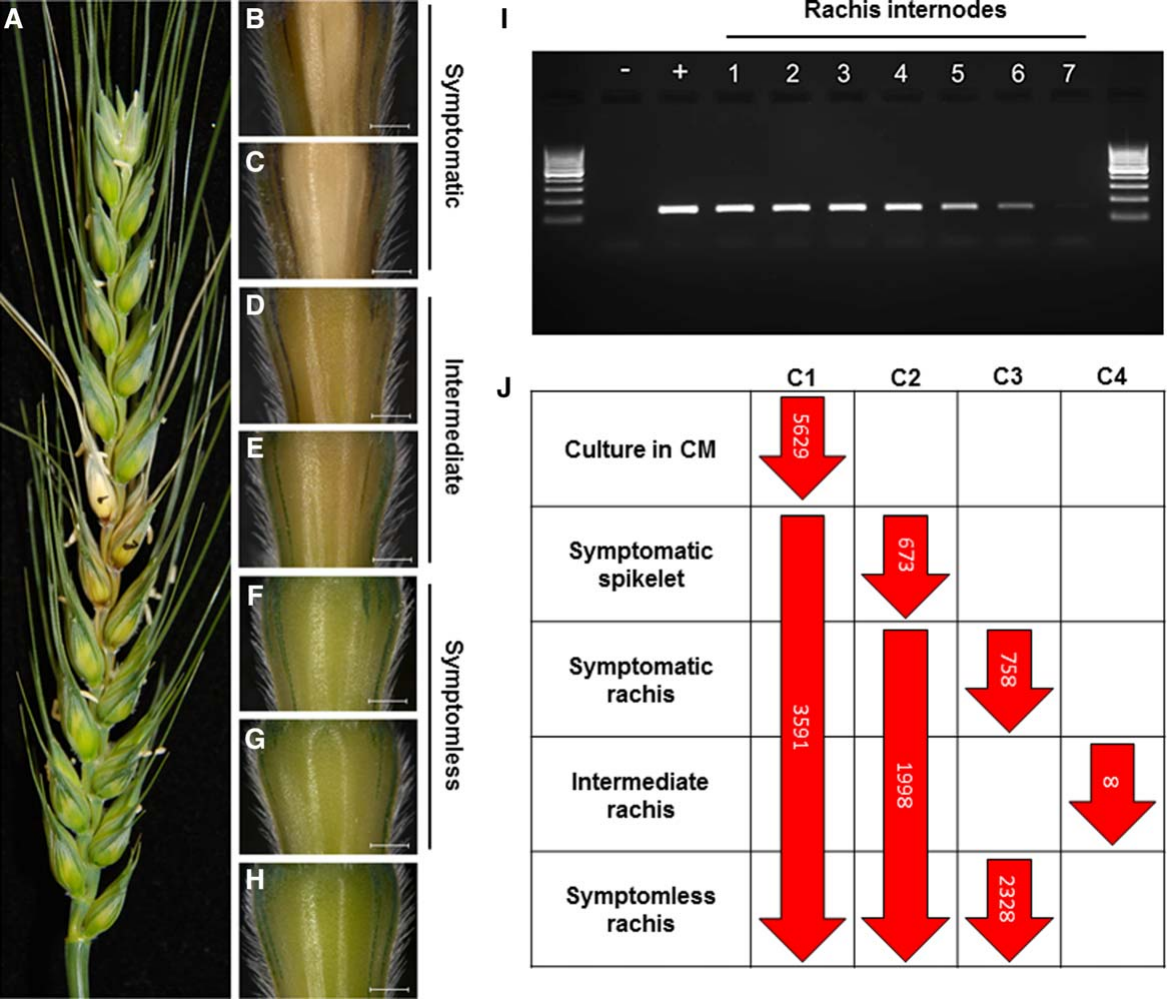
The identification of the *Fusarium graminearum* transcriptome during symptomless and symptomatic wheat head infection. (A) The macroscopic appearance of wheat disease symptoms at 7 days after *F. graminearum* infection. Black dots indicate the two inoculated spikelets. (B–H) The appearance of sequential rachis internodes below the inoculated spikelet utilized in the rachis internode assay to identify the extent of symptomless infection. (I) Diagnostic reverse transcription‐polymerase chain reaction (RT‐PCR) identifies *F. graminearum* infection within rachis internodes 5 and 6, indicating the presence of symptomless infection. The negative control (–), the fungal genomic DNA positive control (+) and the pooled samples from seven sequential rachis internodes below the inoculated spikelet, originating from 15 wheat heads of a representative biological replicate, are presented. (J) The overall expression patterns of the *F. graminearum* transcriptome within different wheat tissues. Comparison C1 used existing Affymetrix data for *F. graminearum* cultured in complete medium (CM) to identify genes which showed increased transcript abundance in either wheat heads or axenic culture. Three additional *in planta* comparisons were used: a rachis versus spikelet comparison (C2), a symptomless versus symptomatic rachis tissue comparison (C3) and a comparison of the intermediate rachis tissue with the mean of the combined symptomless and symptomatic rachis tissues (C4). Significance was determined by applying a Benjamini Hochberg multiple testing correction and a significance level of 0.05.

The 7‐day post‐inoculation (dpi) time point was selected for detailed transcriptomic investigation because of the existence and close proximity of symptomless and symptomatic rachis tissues. The macroscopic symptoms of the rachis internodes and inoculated spikelets showed the clear separation of the symptomless and symptomatic wheat tissues (Fig. 1). Diagnostic polymerase chain reaction (PCR) confirmed the presence of fungal mycelium in the symptomatic and symptomless tissues (Fig. 1). Reverse transcription‐quantitative polymerase chain reaction (RT‐qPCR) analyses of several housekeeping genes revealed that fungal biomass was low within the symptomless, and high within the symptomatic, wheat rachis tissues (Fig. S2A, see Supporting Information). This confirmed the previous microscopic observations which noted a lower level of fungal biomass in the apoplast during symptomless infection, which increased after intracellular colonization of dead plant cells (Brown *et al*., 2010). Phenotypically similar rachis internode pairs were combined from 15 wheat heads per treatment, per independent biological replicate (*n* = 3) and included: (i) symptomless; (ii) intermediate; and (iii) fully symptomatic wheat rachis tissues. The inoculated symptomatic spikelet from the positon at which the infection originated was also included. The infection phase‐specific *F. graminearum* transcriptome was then interrogated using the species‐specific Affymetrix array (Gueldener *et al*., 2006).

### Global analyses reveal the distinct nature of the *F. graminearum* transcriptome during symptomless and symptomatic infection

The first genome‐wide comparison (C1) identified *F. graminearum* genes with increased transcript abundance during plant infection, when compared with *in vitro* culture, revealing those potentially involved in the establishment of infection and growth within the plant host (Fig. 1). This approach has been utilized previously in other *F. graminearum* transcriptomic studies to identify *in planta* expressed genes (Gueldener *et al*., 2006; Lysoe *et al*., 2011; Zhang *et al*., 2012). Probes were filtered on the basis of this comparison to reveal 4062 probes, equivalent to 3591 genes, with significantly (*P* < 0.05) increased transcript abundance during wheat infection (Fig. 1 and File S1, see Supporting Information). In contrast, 5820 probes equivalent to 5629 genes showed increased transcript abundance during culture in complete medium (Fig. 1 and File S1).

Three additional comparisons (C2–C4) identified the *F. graminearum* genes with different spatial temporal expression patterns (Fig. 1 and File S1). The C2 comparison of rachis tissue with inoculated spikelets revealed 2671 genes to be differentially regulated between these different wheat tissue types, identifying 1998 and 673 genes with increased transcript abundance in the rachis or spikelet, respectively. The C3 comparison of symptomless and symptomatic rachis tissues revealed 2328 genes with increased transcript abundance in symptomless tissue and 758 genes with increased transcript abundance in symptomatic wheat tissue. The C4 comparison of rachis tissue in between symptomless and symptomatic tissues, termed intermediate, with the combined mean of the symptomless and symptomatic rachis tissues only identified eight genes to be differentially regulated. Collectively, this implied the following: (i) rachis infection was distinct from that occurring in the spikelet; and (ii) the dynamics of infection and transcription followed a gradient along the continuum of infection within the rachis, where the phenotypically distinct symptomless and symptomatic wheat rachis tissues showed the greatest transcriptional difference.

**Figure 2:**
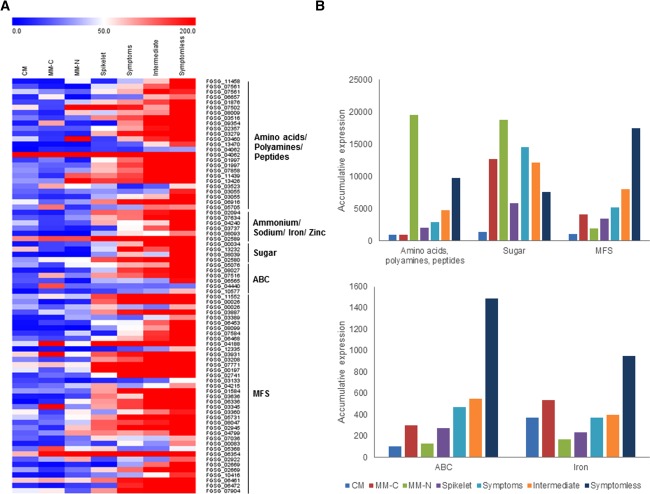
The over‐representation of *Fusarium graminearum* genes coding for putative transmembrane transporters during wheat infection. (A) Heatmap of the expression of putative transporters during *in vitro* growth and wheat infection. (B) The accumulative expression profile of different classes of putative transporters during *in vitro* culture and wheat infection. CM, complete medium; MM‐C, minimal medium without carbon; MM‐N, minimal medium without nitrogen (Gueldener *et al*., 2006).

Insights into the putative functions of these differentially regulated genes were obtained via the identification of over‐represented gene ontology (GO) terms and metabolic pathways (Kyoto Encyclopedia of Genes and Genomes, KEGG) among the Blast2GO (Gotz *et al*., 2008) and MIPS (Mewes *et al*., 2004) annotated genes. There was an over‐representation of *F. graminearum* genes with increased transcript abundance in the rachis, in comparison with the spikelet, which were involved in processes such as transmembrane transport, oxidation–reduction and polysaccharide binding (Table S1, see Supporting Information). However, no over‐represented GO terms were assigned to the genes with increased transcript abundance in the wheat spikelet compared with the rachis. Similarly, the subsequent comparison of symptomless and symptomatic rachis tissues revealed that genes involved in transmembrane transport, in particular putative amino acid, polyamine and metal ion transporters, plus putative ABC and MSF transporters, were increasingly transcribed by *F. graminearum* within symptomless wheat rachis tissue (Table 1 and Fig. 2). In accordance with these observations, the KEGG pathways involved in amino acid metabolism were also highly represented within symptomless wheat rachis tissue (Table S2, see Supporting Information). In addition, genes involved in oxidation–reduction processes, such as cellular redox homeostasis and secondary metabolism, were increasingly transcribed in symptomless wheat rachis tissue (Table 1). By contrast, within the symptomatic wheat tissue, genes involved in polysaccharide utilization and protein translation showed increased transcript abundance, in comparison with symptomless wheat rachis tissue (Table 1). This was reflected in the increased representation of starch metabolism and glycan degradation, plus glyoxylate and dicarboxylate metabolism, among the KEGG pathways highly represented in symptomatic rachis tissue (Table S2). Therefore, the predicted metabolic state of *F. graminearum* appears to be distinct within symptomless and symptomatic wheat rachis tissues.

**Table 1:** The over‐represented gene ontology (GO) terms assigned to the *Fusarium graminearum* genes with increased transcript abundance within symptomless (A) or symptomatic (B) wheat rachis tissue.

	GO‐ID	Term	Category	FDR	*P* value	Genes
A	0055085	Transmembrane transport	BP	3.91E‐04	8.07E‐08	122
	0016021	Integral component of membrane	CC	5.20E‐04	2.81E‐07	154
	0055114	Oxidation–reduction process	BP	1.69E‐03	1.74E‐06	167
	0004497	Monooxygenase activity	MF	7.05E‐03	8.73E‐06	40
B	0003735	Structural constituent of ribosome	MF	3.69E‐24	7.61E‐28	48
	0005975	Carbohydrate metabolic process	BP	1.79E‐15	2.59E‐18	72
	0006412	Translation	BP	4.88E‐15	8.06E‐18	57
	0005576	Extracellular region	CC	1.48E‐04	6.11E‐07	16
	0030248	Cellulose binding	MF	7.10E‐04	3.67E‐06	8
	0046556	α‐l‐Arabinofuranosidase activity	MF	7.31E‐04	4.06E‐06	5
	0046373	l‐Arabinose metabolic process	BP	1.82E‐03	1.21E‐05	4
	0015935	Small ribosomal subunit	CC	3.68E‐03	2.81E‐05	7
	0045493	Xylan catabolic process	BP	2.12E‐02	2.00E‐04	6
	0016702	Oxidoreductase activity, acting on single donors with incorporation of molecular oxygen, incorporation of two atoms of oxygen	MF	2.12E‐02	2.00E‐04	6
	0030245	Cellulose catabolic process	BP	2.12E‐02	2.06E‐04	3
	0008810	Cellulase activity	MF	3.49E‐02	3.67E‐04	4

BP, biological process; CC, cellular component; MF, molecular function.

Consequently, the following comparative analyses primarily focused on the phenotypically and transcriptionally distinct extremities, i.e. the symptomless and symptomatic wheat rachis tissues. The *F. graminearum* genes with increased transcript abundance within infected symptomless or symptomatic wheat rachis tissue were mapped to the four *F. graminearum* chromosomes, together with single nucleotide polymorphism (SNP) density and recombination frequencies (Cuomo *et al*., 2007), using the OmniMapFree software (Antoniw *et al*., 2011). This confirmed that 45% and 47% of genes with increased transcript abundance in symptomless and symptomatic wheat rachis tissue, respectively, resided in hotspots exhibiting a high level of chromosomal recombination (Fig. S3, see Supporting Information).

### Analyses of characterized toxic and non‐toxic secondary metabolite gene clusters reveal DON mycotoxin induction within symptomless wheat tissue

**Figure 3:**
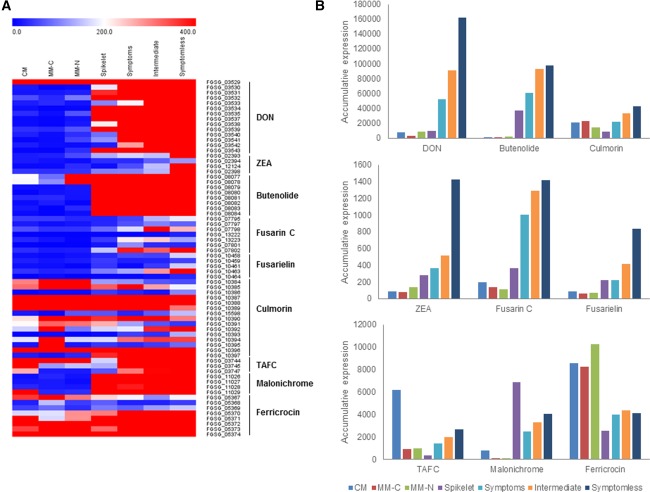
The induction of *Fusarium graminearum* genes coding for characterized toxic and non‐toxic secondary metabolites during wheat infection. (A) Heatmap of the expression of the genes within characterized secondary metabolite gene clusters (Sieber *et al*., 2014) during *in vitro* growth and wheat infection. (B) The accumulative expression profile of nine well‐characterized secondary metabolite gene clusters during *in vitro* growth and wheat infection. DON, deoxynivalenol; TAFC, triacetyl fusarinine C; ZEA, zearalenone. CM, complete medium; MM‐C, minimal medium without carbon; MM‐N, minimal medium without nitrogen (Gueldener *et al*., 2006).

The aforementioned Blast2GO analyses revealed that genes encoding for polyketide synthases and cytochrome P450s, involved in oxidation–reduction reactions and the biosynthesis of secondary metabolites, showed increased transcript abundance in symptomless wheat rachis tissue. A detailed bioinformatics analysis has previously identified 67 putative and characterized secondary metabolite gene clusters in *F. graminearum*, including 13 clusters with known metabolites (Sieber *et al*., 2014). The expression of the gene clusters responsible for the production of the characterized secondary metabolites was assessed (Fig. 3A and File S2, see Supporting Information). *Fusarium graminearum* produces at least two classes of toxic and non‐toxic secondary metabolites, which are known to be required for *F. graminearum* virulence on wheat, namely the mycotoxin DON and the siderophores TAFC and malonichrome (Brown and Hammond‐Kosack, 2015; Oide *et al*., 2006, 2015; Proctor *et al*., 1995).

The *TRI* genes that encode for biosynthetic enzymes involved in the production of the DON mycotoxin showed a dramatic increase in transcript abundance within symptomless wheat tissue (Fig. 3B). Accordingly, the two transcriptional inducers of the *TRI* cluster and DON biosynthesis, *TRI6* and *TRI10*, both also showed increased transcript abundance in symptomless tissues. This strongly suggests that the maximal expression of the *TRI* genes in symptomless wheat tissue is a consequence of *Tri6‐ and Tri10*‐mediated gene induction. Conversely, *TRI* gene expression was reduced in the later symptomatic tissue. The *Tri6 and Tri10* transcription factors have been shown to regulate DON production and are required for full virulence (Nasmith *et al*., 2011; Seong *et al*., 2009; Son *et al*., 2011). In addition, in the absence of the *TRI5* terpene synthase encoding gene required for DON production, *F. graminearum* loses the ability to establish symptomless infection and the wheat plant mounts an enhanced defensive response (Cuzick *et al*., 2008). The Affymetrix expression data for the *TRI* cluster were independently validated via RT‐qPCR. This analysis showed the low level of γ‐actin and β‐tubulin expression in symptomless tissues, reflecting the low level of fungal biomass, and the higher level of expression within symptomatic wheat tissues, correlating with the increase in fungal biomass, which is in accordance with previous histological evidence (Brown *et al*., 2010). This analysis also confirmed the increased expression of *TRI4* and *TRI5* within symptomless wheat tissue (Fig. S2B), validating the findings of the Affymetrix investigation. Therefore, the *TRI* genes involved in DON biosynthesis are highly induced in symptomless tissues, whereas DON is required for the establishment of symptomless infection.

*Fusarium graminearum* can also produce other mycotoxins, including butenolide (which has low pharmacological toxicity), culmorin (which is phytotoxic at high concentrations and is also described as having antifungal properties) and fusarin C (which is a possible carcinogen) (Sieber *et al*., 2014). Similar to the *TRI* cluster, the butenolide and culmorin gene clusters showed increased transcript abundance in symptomless wheat tissue, whereas the clusters involved in the production of the oestrogenic metabolite zearalenone, plus fusarielin and fusarin C, were induced, but at a lower absolute level (Fig. 3B). Nonetheless, the individual disruption of the ability to produce these mycotoxins has no impact on *F. graminearum* virulence on wheat (Sieber *et al*., 2014).

The gene cluster involved in the biosynthesis of the major iron‐scavenging extracellular siderophore TAFC showed a moderate increase in transcript abundance in symptomless relative to symptomatic wheat rachis tissue (Fig. 3B). Similarly, genes encoding for siderophore transporters showed increased transcript abundance in symptomless tissues (Fig. 2C). The gene cluster involved in the production of an additional extracellular siderophore, malonichrome, also showed a moderate increase in transcript abundance in symptomless rachis tissue, but was most highly expressed within the inoculated fully symptomatic spikelet (Fig. 3B). In contrast, the gene cluster responsible for the biosynthesis of the intracellular siderophore, ferricrocin, was constantly expressed at a higher level during *in vitro* culture (Fig. 3B). In *F. graminearum*, the absence of *NPS6* or *NPS1*, which are required for the production of TAFC and malonichrome, respectively, results in a loss in virulence and, in the case of *NPS6*, the wheat plant subsequently mounts an enhanced defensive response (Oide *et al*., 2006, 2015). In contrast, the loss of *NPS2*, responsible for ferricrocin biosynthesis, has no impact on virulence (Oide *et al*., 2015).

### Analyses of putative secondary metabolite gene clusters identifies additional clusters induced within symptomless wheat tissue

**Figure 4:**
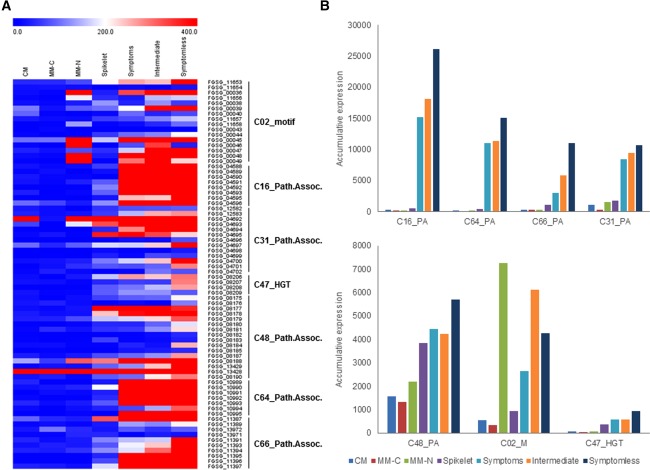
The induction of additional putative secondary metabolite gene clusters in *Fusarium graminearum* during wheat infection. (A) Heatmap of the expression of the genes within seven putative and uncharacterized secondary metabolite gene clusters (Sieber *et al*., 2014) during *in vitro* growth and wheat infection. HGT, horizontal gene transfer. (B) The accumulative expression profile of the seven putative secondary metabolite gene clusters during *in vitro* growth and wheat infection. CM, complete medium; MM‐C, minimal medium without carbon; MM‐N, minimal medium without nitrogen (Gueldener *et al*., 2006).

In addition to the known metabolites produced by *F. graminearum*, an additional 54 putative secondary metabolite gene clusters have been predicted (Sieber *et al*., 2014). The expression of the gene clusters responsible for these putative secondary metabolites was evaluated (Fig. 4A and File S2). As a result of their transcriptional induction *in planta*, in pre‐existing whole wheat and barley head experiments, Sieber *et al*. (2014) proposed three novel clusters to potentially be involved in plant infections, namely C16, C62 and C64, whereas cluster C02 contained a conserved binding motif in the promoter of each gene, and cluster C47 showed evidence of a horizontal gene transfer event. The present study corroborated some of these findings and identified additional putative clusters to be highly expressed in symptomless wheat tissue (Fig. 4B). Clusters C16, C31, C64 and C66 showed increased transcript abundance within symptomless wheat tissue. Clusters C02, C47 and C48 showed increased transcript abundance in symptomless wheat tissue, but at a lower level, whereas C02 was also induced by nitrogen starvation. In total, 80% of genes within the 67 characterized and putative secondary metabolite clusters, which showed increased transcript abundance in symptomless wheat tissue, predominantly localized to hotspots with high levels of chromosomal recombination (Fig. S3).

### Analyses of putative secreted fungal effectors reveal distinct groups to be induced within either symptomless or symptomatic wheat tissues

**Figure 5:**
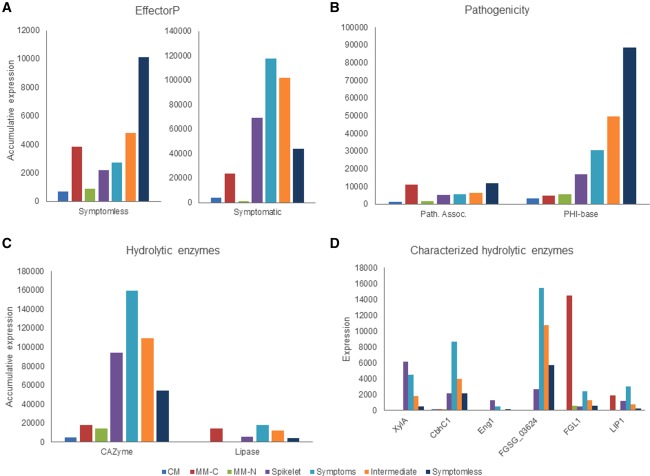
The expression of various categories of *Fusarium graminearum* genes previously associated with virulence. (A) The accumulative expression of transcripts encoding for putative secreted effectors with increased transcript abundance in either symptomless or symptomatic wheat tissue. (B) The accumulative expression of PHI‐base genes, and those previously defined as being pathogenicity associated, during *in vitro* culture and wheat infection. (C) The two‐step regulation of hydrolytic enzyme production by *F. graminearum* during wheat infection. The accumulative expression of 50 putative carbohydrate‐active enzyme (CAZyme) and six lipase encoding genes throughout *in vitro* culture and wheat infection. This revealed increased CAZyme and lipase transcript abundance *in planta* versus *in vitro* culture, and the highest accumulative CAZyme abundance within symptomatic wheat tissue. (D) The expression profiles of four characterized CAZymes, which have been proven to contribute to virulence (Urban *et al*., 2015), and two characterized lipases peak in symptomatic wheat tissue. CM, complete medium; MM‐C, minimal medium without carbon; MM‐N, minimal medium without nitrogen (Gueldener *et al*., 2006).

A stringent bioinformatics analysis previously defined the *F. graminearum* secretome to contain 574 proteins, revealing the pathogen's hydrolytic arsenal and candidate fungal effector repertoire (Brown *et al*., 2012). In total, 66% of the refined secretome encoding genes which showed increased transcript abundance in symptomless wheat tissue localized to the known hotspots of chromosomal recombination (Fig. S3). EffectorP software (Sperschneider *et al*., 2016) was subsequently used to identify the putative *F. graminearum* effectors within the refined secretome prediction. This approach identified 39 putative effectors that were induced during wheat infection, 18 of which showed increased transcript abundance within symptomless wheat tissue, and 21 of which showed increased transcript abundance within symptomatic wheat tissue (Fig. 5A, Table 2 and File S2). Only a single protein within the putative effectors with increased transcript abundance in symptomless wheat tissue, FGSG_01688, possessed functional annotation, containing a predicted CyanoVirin‐N homology domain (PFAM08881). Among these unannotated putative effector proteins, the most dramatic transcriptional modulations included several small cysteine‐rich secreted proteins (<100 amino acids, >8% C content in the predicted mature peptide), namely FGSG_00847, FGSG_02378, FGSG_15448 and FGSG_15469. In the symptomatic wheat tissue, five putative effectors with increased transcript abundance possessed functional annotation, including three CAZymes [FGSG_03624 (PFAM00457), FGSG_04848 (PFAM00657) and FGSG_10670 (PFAM01083)], plus a chloroperoxidase FGSG_03436 (PFAM01328) and a hypothetical protein FGSG_03911 with a phospholipase domain (PFAM09056). None of the differentially regulated putative effectors have been described previously as showing signatures of being exposed to diversifying selection pressure (Sperschneider *et al*., 2015). Therefore, *F. graminearum* coordinates the spatial temporal induction of different groups of putative effectors that show either increased transcript abundance in symptomless tissue or increased transcript abundance in symptomatic tissue.

**Table 2:** The 39 putative secreted *Fusarium graminearum* effector protein encoding genes with increased transcript abundance within symptomless (A) or symptomatic (B) wheat rachis tissue.

	FGSG_ID	EffectorP probability	No. amino acids	WolfPsort score	MIPS annotation	PFAM	No. Cys	% Cys	RADAR repeats
A	00009	0.648	189	extr=26	Hypothetical protein	–	4	2.12	2
	00029	0.997	158	extr=21	Conserved hypothetical protein	–	11	6.96	–
	00847	0.512	48	extr=23	Conserved hypothetical protein	–	4	8.33	–
	01688	1	222	extr=18	Hypothetical protein	08881	12	5.41	2
	02309	0.975	130	extr=26	Conserved hypothetical protein	–	8	6.15	–
	02378	0.996	99	extr=26	Conserved hypothetical protein	–	8	8.08	–
	02674	0.986	100	extr=24	Conserved hypothetical protein	–	6	6	2
	03035	0.965	132	extr=21	Conserved hypothetical protein	–	4	3.03	2
	03463	0.869	157	extr=18	Conserved hypothetical protein	–	0	0	–
	04624	0.963	200	extr=19	Conserved hypothetical protein	–	9	4.5	–
	04699	0.985	129	extr=22	Hypothetical protein	–	6	4.65	–
	05341	0.848	173	extr=19	Conserved hypothetical protein	–	5	2.89	2
	05841	1	89	extr=22	Hypothetical protein	–	8	8.99	–
	07221	0.999	96	extr=22	Hypothetical protein	–	4	4.17	–
	07829	0.944	167	extr=27	Conserved hypothetical protein	–	3	1.8	–
	12214	1	79	extr=27	Hypothetical protein	–	10	12.66	2
	15448	1	71	extr=19	Conserved hypothetical protein	–	8	11.27	–
	15469	0.999	74	extr=21	Conserved hypothetical protein	–	6	8.11	2
B	02685	0.998	128	extr=24	Conserved hypothetical protein	–	8	6.25	2
	03334	0.806	132	extr=27	Conserved hypothetical protein	–	10	7.58	2
	03436	0.665	213	extr=23	Related to chloroperoxidase	01328	1	0.47	3
	03584	0.999	157	extr=21	Conserved hypothetical protein	–	2	1.27	2
	03599	0.97	77	extr=21	Conserved hypothetical protein	–	10	12.99	3
	03600	0.942	166	extr=24	Conserved hypothetical protein	–	8	4.82	2
	03624	0.776	209	extr=26	Probable endo‐1,4‐β‐xylanase A precursor	00457	2	0.96	2
	03748	0.553	236	extr=23	Conserved hypothetical protein	–	4	1.69	2
	03911	0.947	167	extr=24	Conserved hypothetical protein	09056	4	2.4	2
	04074	0.955	171	extr=25	Conserved hypothetical protein	–	6	3.51	–
	04583	0.967	130	extr=24	Conserved hypothetical protein	–	4	3.08	–
	04745	1	74	extr=22	Related to antifungal protein	–	6	8.11	2
	04848	0.861	237	extr=19	Probable rhamnogalacturonan acetylesterase precursor	00657	2	0.84	2
	07899	0.886	197	extr=21	Conserved hypothetical protein	–	10	5.08	–
	07921	0.575	206	extr=23	Conserved hypothetical protein	–	3	1.46	2
	10670	0.952	207	extr=21	Probable acetylxylan esterase precursor	01083	10	4.83	2
	11033	0.791	90	extr=26	Conserved hypothetical protein	–	2	2.22	2
	11647	1	100	extr=22	Hypothetical protein	–	8	8	–
	12081	0.799	120	extr=25	Hypothetical protein	–	1	0.83	–
	15251	0.997	47	extr=23	Conserved hypothetical protein	–	6	12.77	–
	15437	1	53	extr=25	Hypothetical protein	–	8	15.09	–

Cys, cysteine; RADAR, Rapid Automatic Detection and Alignment of Repeats.

### The induction of other types of pathogenicity genes within symptomless wheat tissue

A recent comparative genomic study of three *Fusarium* species identified rapidly evolving pathogenicity‐associated genes, i.e. those that encode proteins with homologues in other fungal pathogens, but are absent from non‐pathogenic fungi (Sperschneider *et al*., 2015), thereby identifying genes more likely to be involved in pathogenesis. Again, the abundance of transcripts and the diversity of these pathogenicity‐associated genes increased within symptomless wheat tissue (Fig. 5B and File S2). These 27 identified pathogenicity‐associated genes included four hypothetical metal ion binding proteins (FGSG_05017, FGSG_05020, FGSG_07759 and FGSG_12405) and three secreted proteins, including a putative nodulin precursor (FGSG_03550) and two unannotated proteins (FGSG_04521 and FGSG_05947). All 27 identified pathogenicity‐associated proteins have been defined previously as being exposed to diversifying selection (Sperschneider *et al*., 2015). Therefore, the number of pathogenicity‐associated genes with increased transcript abundance in symptomless wheat tissue, coupled with their distribution solely within the genomes of fungal pathogens, and their exposure to diversifying evolution pressure, supports the hypothesis that these genes may be important to the establishment of *F. graminearum* wheat infection.

The Pathogen–Host Interactions database (www.PHI-base.org) contains ∼7000 curated interactions obtained from the peer‐reviewed literature, and provides phenotypic data associated with specific genetic mutations in a pathogen and their impact on virulence (Urban *et al*., 2015). Within PHI‐base, 956 *F. graminearum* single‐gene mutations have been curated with phenotypic data, including 188 with loss of pathogenicity/reduced virulence, two with increased virulence, 73 with lethality and 693 with unaffected virulence. In total, 142 PHI‐base genes exhibited increased transcript abundance during wheat infection, including 111 with increased abundance during symptomless infection and 31 with increased abundance during symptomatic infection. However, when disrupted or deleted, only 15 of these PHI‐base genes induced during wheat infection were reported to have an impact on virulence (Table 3 and File S2). The accumulative expression of the PHI‐base genes required for virulence was higher in symptomless wheat tissue (Fig. 5B). Amongst the 13 PHI‐base genes required for virulence with increased transcript abundance in symptomless wheat tissue were genes involved in DON and Culmorin biosynthesis (*TRI5, TRI14,* and Clm2), the regulation of DON production (*VELB*), and genes involved in fundamental processes, such as ergosterol biosynthesis (*ERG5*), histone deacetylation (*HDF1*) and the cell cycle (*CID1* and *CAK1*). Conversely, the two PHI‐base genes with increased transcript abundance in symptomatic wheat tissue, which conferred a reduced virulence phenotype, encoded for a histone acetyltransferase and a putative Dsk1 protein kinase (Table 3). This PHI‐base informed analysis has revealed that the majority of the 188 genes formally shown to be required for disease formation by *F. graminearum* are not significantly induced during early rachis colonization.

**Table 3:** The *Fusarium graminearum* Pathogen–Host Interactions database (PHI‐base) genes with a proven role in virulence on wheat heads which showed increased transcript abundance within either symptomless (A) or symptomatic (B) wheat tissue at 7 days post‐inoculation.

	PHI‐ID	FGSG_ID	Gene	Description	Phenotype
A	3037	01959	*FgERG5*	Cytochrome P450 involved in ergosterol biosynthesis	Reduced virulence
	1169	04324	*HDF1*	Histone deacetylase	Reduced virulence
	2418	04355	*CID1*	C‐type cyclins	Reduced virulence
	1192	04947	*CAK1*	Cyclin‐dependent protein kinase	Reduced virulence
	1805	08028	*GzZC120*	Transcription factor	Reduced virulence
	1801	08182	*GzZC116*	Transcription factor	Reduced virulence
	1425	11416	*GzC2H093*	Transcription factor	Reduced virulence
	4658	00500	*FgHXK1*	Hexokinase	Reduced virulence, reduced DON
	2427	01362	*FgVelB*	Velvet complex	Reduced virulence, reduced DON
	4243	03537	*TRI5*	Trichodiene synthase	Reduced virulence, reduced DON
	525	03543	*TRI14*	Trichothecene biosynthesis	Reduced virulence, reduced DON
	2393	00007	*Clm2* *	Cytochrome P450 involved in culmorin biosynthesis	Increased virulence, increased DON
B	3138	02040	*ELP3*	Elongator complex	Reduced virulence
	1193	02795	*Sky1*	Dis1‐suppressing protein kinase dsk1	Reduced virulence

DON, deoxynivalenol.

*FGSG_00007 reclassified as FGSG_17598/Clm2 Bahadoor *et al*., 2016

### The distinct transcriptional profile of CAZymes and lipases in symptomless and symptomatic wheat tissue

The *F. graminearum* genome encodes 109 putative CAZymes, which are predicted, via their similarity to functionally characterized enzymes of related fungi, to participate in the breakdown of plant biomass into utilizable saccharides (Brown and Hammond‐Kosack, 2015; Brown *et al*., 2014). In comparison with *in vitro* culture in nutrient‐rich medium, 50 putative secreted CAZymes showed increased transcript abundance during the infection of symptomless wheat rachis tissue. However, in wheat tissue which had developed disease symptoms, CAZyme transcript abundance significantly increased to a higher level than in symptomless wheat tissue (Fig. 5C and File S2). This confirmed the previously observed over‐representation of biological processes involved in polysaccharide breakdown among the genes with increased transcript abundance in symptomatic wheat tissue in comparison with symptomless tissue (Table 1). Several CAZymes in *F. graminearum* have been proven to be required for full virulence on wheat, namely CbhC1, Eng1 and XylA (Sperschneider *et al*., 2016; Zhang *et al*., 2012), whereas the xylanase FGSG_3624 has also been shown to induce host cell death (Sella *et al*., 2013). The examination of these functionally characterized CAZymes revealed the highest level of transcript abundance in symptomatic wheat tissue (Fig. 5D). Therefore, the complex organization of CAZyme induction in *F. graminearum* during wheat infection appears to be regulated by two mechanisms: first, the induction of limited CAZyme expression in symptomless wheat tissue; second, the augmentation of CAZyme expression during the development of disease symptoms.

The *F. graminearum* genome encodes 15 putative secreted lipases (Brown *et al*., 2012). Similar to CAZyme expression, six lipases (FGSG_05906, FGSG_12119, FGSG_02360, FGSG_03530, FGSG_03612 and FGSG_04848) showed increased transcript abundance in symptomless wheat tissue when compared with *in vitro* culture in nutrient‐rich medium (Fig. 5C and File S2). Again, lipase transcript abundance was highest in symptomatic wheat rachis tissue. Two functionally characterized secreted lipases in *F. graminearum*, namely *LIP1* and *FGL1*, have been shown previously to be strongly induced by wheat‐germ oil (Bluemke *et al*., 2014; Feng *et al*., 2005). The absence of either lipase has an impact on the lipolytic activity of *F. graminearum*, whereas only *FGL1* has been shown to be required for full virulence (Bluemke *et al*., 2014; Feng *et al*., 2005). During infection, both characterized lipases showed their highest transcript abundance within symptomatic wheat rachis tissue (Fig. 5D). *FGL1* has previously been found to be highly induced by carbon starvation (Gueldener *et al*., 2006). Therefore, lipases, as with the CAZymes described above, were found to be transcriptionally induced throughout wheat infection, and their expression was maximal in symptomatic wheat tissue when the fungal pathogen was associated with dead wheat cells.

### Comparison with other *F. graminearum* Affymetrix datasets reveals that different phases of infection co‐exist within a single time point, which are reminiscent of both early and late infection

**Figure 6:**
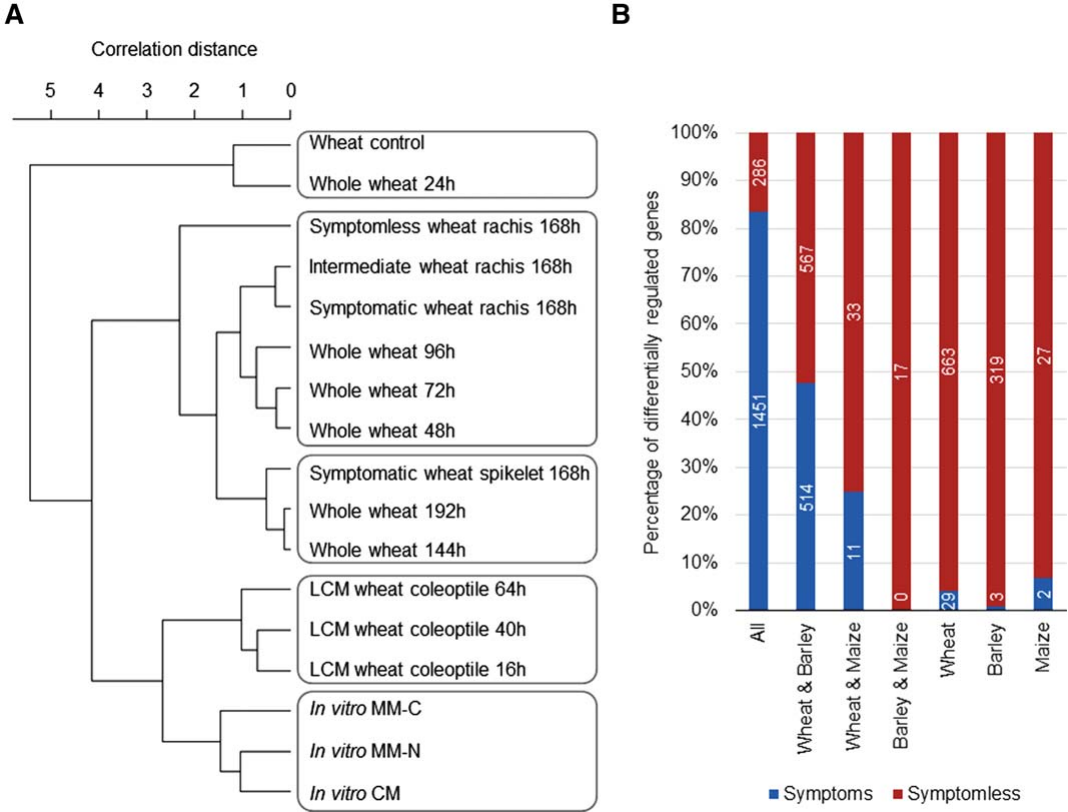
Genome‐wide comparative analyses of *Fusarium graminearum* gene expression during wheat, barley and maize infection. (A) Dendrogram of the genome‐wide correlation distance (complete linkage) between the presented *F. graminearum* infection phase‐specific transcriptome isolated from symptomless and symptomatic wheat tissues at 7 days post‐infection (dpi) (168 h), with pre‐existing *in vitro* (Gueldener *et al*., 2006) and *in planta* datasets, including whole wheat heads (complete time course) and laser‐capture micro‐dissection (LCM) wheat coleoptiles (Lysoe *et al*., 2011; Zhang *et al*., 2012) (available from PlexDB). It should be noted that the transcriptional profiles of early and late wheat head infection are simultaneously linked to the phenotypically distinct symptomless and symptomatic tissues within the single 7‐dpi time point. CM, complete medium; MM‐C, minimal medium without carbon; MM‐N, minimal medium without nitrogen. (B) The distribution of the previously reported host species‐specific and commonly expressed *F. graminearum* genes (Soanes *et al*., 2008) within the genes with increased transcript abundance in the symptomless and symptomatic wheat rachis tissue at 7 dpi. The increased percentage of reported host‐specific genes in symptomless wheat tissue should be noted.

The current *F. graminearum* wheat infection phase‐specific transcriptome was compared with pre‐existing Affymetrix datasets for *F. graminearum* cultured *in vitro* (Gueldener *et al*., 2006) and in association with wheat, including a whole wheat head experiment (Lysoe *et al*., 2011) and LCM samples obtained from juvenile wheat coleoptiles (Zhang *et al*., 2012) (Fig. 6A). The whole wheat head 1‐dpi time point and the wheat negative control from the study of Lysoe *et al*. (2011) were closely related (0.88). In contrast, the *F. graminearum* transcriptome associated with the symptomless wheat rachis tissue at 7 dpi was the most distinct treatment amongst all the wheat datasets, and showed its highest correlation with the early 2‐dpi transcriptome isolated from whole wheat heads (0.90). Conversely, the transcriptome isolated from the symptomatic wheat rachis tissue at 7 dpi correlated with the 4‐dpi (0.95) transcriptome from whole wheat heads, i.e. after the development of FHB disease symptoms (Lysoe *et al*., 2011). The fully symptomatic spikelet at 7 dpi, which represented the origin of infection, correlated (0.95–0.96) with the final time points from the whole wheat head infection (6–8 dpi). These comparative analyses suggest that, at a single time point, within the entire wheat head, different phases of infection co‐exist, which are reminiscent of both early and late infection. In addition, these analyses highlight the differences in the individual bioassays and the impact of wheat tissue type on the fungal transcriptome, as shown by the reduced correlation of the wheat coleoptile transcriptome with the wheat head and rachis studies.

### Comparison of host species‐specific and commonly expressed genes redefines plant host transcriptional boundaries for *F. graminearum*

An analysis of the *F. graminearum* transcriptome isolated from infected whole wheat, barley and maize heads previously described the host species‐specific *F. graminearum* transcriptome (Harris *et al*., 2016). The expression of these different subsets of host species‐specific and commonly expressed *F. graminearum* genes was assessed in the present transcriptome data obtained from symptomless and symptomatic wheat tissue (Fig. 6B). By comparison of these datasets with the spatial temporal‐specific *F. graminearum* wheat transcriptome generated in this study, we have identified 1737 genes expressed in all three hosts, 1081 genes expressed only in wheat and barley, 44 genes expressed only in wheat and maize, 17 genes expressed only in barley and maize, plus 692, 322 and 29 genes specifically expressed in wheat, barley or maize, respectively (File S3, see Supporting Information).

From the 1737 *F. graminearum* genes that were expressed in all three host cereal species, 1451 genes showed increased transcript abundance in symptomatic wheat tissue. These included 88 genes that encoded for putative secreted proteins primarily involved in plant cell degradation, such as CAZymes, lipases and proteases, which showed their highest transcript abundance in symptomatic wheat tissue (File S3). Conversely, only 286 *F. graminearum* genes that were expressed in all three host plants showed increased transcript abundance in symptomless wheat tissue. Interestingly, these included seven *TRI* genes (File S3). A similar number of *F. graminearum* genes, which were reported to be specifically expressed during wheat and barley infection, showed increased transcript abundance in either symptomless (567 genes) or symptomatic (514 genes) wheat tissue. These included 42 genes that encoded for putative secreted proteins with increased transcript abundance in symptomatic wheat tissue, for example multiple CAZymes, lipases and two necrosis‐inducing peptides (File S3). Conversely, among the wheat and barley‐specific genes with increased transcript abundance in symptomless wheat tissue, 28 encoded for putative secreted proteins, including two cellobiose dehydrogenases (PF00732), potentially involved in the oxidative breakdown of polysaccharides (Kracher *et al*., 2016).

The genes that had previously been identified to be expressed in a single host‐specific manner, i.e. wheat, barley or maize specific, were predominantly identified in the present study to show increased transcript abundance within symptomless wheat tissue (File S3). The wheat‐specific genes with increased transcript abundance in symptomless wheat tissue included five PHI‐base entries for a fungal Zn(2)–Cys(6) binuclear cluster domain (PF00172) containing transcription factors, three of which, when explored for function, caused reduced *F. graminearum* virulence on wheat (Son *et al*., 2011). Other wheat‐specific genes with increased transcript abundance in symptomless wheat tissue included 30 putative secreted proteins, such as two PAN‐1 domain (PF00024)‐containing proteins, which have been commonly identified in the predicted secretomes of other phytopathogenic fungal species and mediate protein–protein and/or protein–carbohydrate interactions (Soanes *et al*., 2008).

Among the previously reported barley‐specific genes, which showed increased transcript abundance in symptomless wheat tissue, were five PHI‐base entries for transcription factors containing various domains, namely fungal Zn(2)–Cys(6) binuclear cluster (PF00172), bZIP (PF00170), zinc knuckle (PF00098) and Gti1_Pac2 (PF09729) domains. Only Gti1_Pac2, when tested for function, caused reduced *F. graminearum* virulence on wheat (Son *et al*., 2011). Sixteen putative secreted proteins were identified within the previously reported barley‐specific genes to show increased transcript abundance in symptomless wheat tissue. These included a cyanovirin‐N domain (PF08881)‐containing protein, which has been frequently identified as a candidate fungal effector in other phytopathogenic ascomycete fungi, namely *Sclerotinia sclerotiorum* (Guyon *et al*., 2014; Heard *et al*., 2015) and *Colletotrichum higginsianum* (Kleemann *et al*., 2012), and FGSG_04521, which shows hallmarks of being exposed to diversifying selection pressure (Sperschneider *et al*., 2015). Finally, only a limited number of the previously reported maize‐specific genes were found to show increased transcript abundance in symptomless wheat tissue. These included two PHI‐base gene entries predicted to possess a helix–loop–helix DNA‐binding domain (PF00010) and a putative MFS monocarboxylate transporter, both of which, when functionally tested, had no influence on *F. graminearum* virulence on wheat. Two NmrA‐like proteins containing the PF05368 domain and a single putative secreted protein (FGSG_12554), also within the maize‐specific genes, were found to show increased transcript abundance in symptomless wheat tissue. Overall, these inter‐host species comparisons reveal that the plant host boundaries initially established (Harris *et al*., 2016) will continue to be further refined as more datasets are included in these types of analyses.

## Discussion

Globally, FHB has a drastic impact on sustainable and safe cereal production. This is because of the lack of good crop resistance and the pathogen's inherent fungicide insensitivity. The discovery of symptomless *F. graminearum* wheat infection has implications for the development and evaluation of FHB control strategies (Brown *et al*., 2010, 2011). In addition, the prolonged interaction between *F. graminearum* hyphae and live wheat cells does not resemble the three classical definitions: biotrophy, hemibiotrophy and necrotrophy (Brown *et al*., 2010, 2011). Similarly, a covert penetration phase of *F. graminearum* wheat coleoptile infection has been identified (Zhang *et al*., 2012). Hence, this interaction between live fungal and plant cells strongly questions the previous definition of *F. graminearum* as a classical necrotroph that kills host cells ahead of infection.

**Figure 7:**
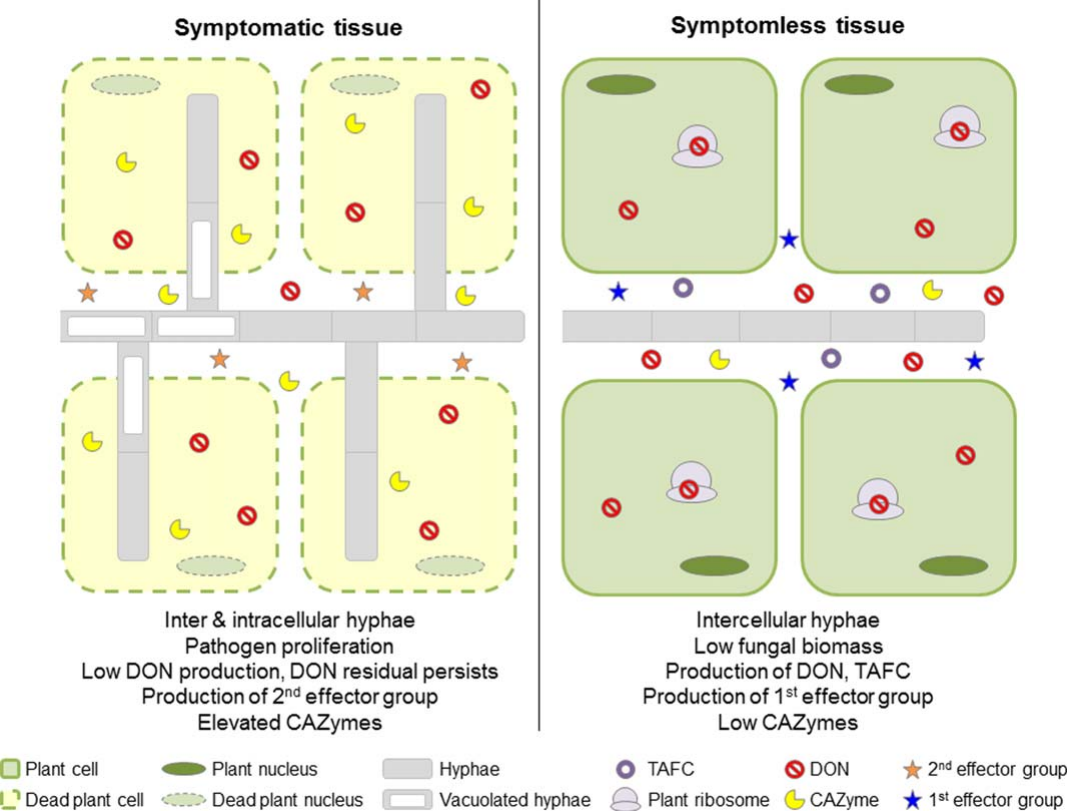
A spatial temporal model for *Fusarium graminearum* infection of wheat floral tissue. This model depicts the spatial temporal transcriptional regulation of characterized and putative virulence strategies deployed in the symptomless and symptomatic wheat tissue of a compatible interaction. CAZyme, carbohydrate‐active enzyme; DON, deoxynivalenol; TAFC, triacetyl fusarinine C.

The present study describes the utilization of the novel rachis internode assay, performed using intact flowering wheat plants, to accurately identify and interrogate the *F. graminearum*–wheat infection process in symptomless and symptomatic wheat tissues. This transcriptomic study of the natural pathogenic interaction complements the histological identification of symptomless wheat infection (Brown *et al*., 2010, 2011), and provides the first insights into the coordinated spatial temporal expression of multiple known and hypothesized virulence strategies deployed by *F. graminearum* during wheat rachis colonization (Fig. 7).

The secretion of at least two types of secondary metabolites, namely DON and two siderophores, is already known to be required for the development of full FHB symptoms (Oide *et al*., 2006; Proctor *et al*., 1995). In this study, the *TRI* genes encoding the biosynthetic enzymes and transcriptional activators for DON production were dramatically induced in symptomless wheat tissue. Plant‐derived nitrogen compounds, such as ornithine and putrescine from the polyamine biosynthetic pathway, are potent inducers of DON (Gardiner *et al*., 2009, 2010). The elicitation of plant polyamine biosynthesis is already known to be an early response to *F. graminearum* infection, which results in polyamine accumulation prior to DON production (Gardiner *et al*., 2009, 2010). Interestingly, in symptomless wheat tissue, *F. graminearum* induced the transcription of various putative amino acid and polyamine transporters. Therefore, in symptomless wheat tissue, the apoplastically dwelling *F. graminearum* hyphae may utilize the recognition and import of wheat amino acids and polyamines as a trigger for DON production. The mycotoxin DON binds to the small ribosomal subunit, inhibiting protein translation in eukaryotes (Ehrlich and Daigle, 1987). The absence of a key enzyme in DON biosynthesis, trichodiene synthase (*Tri5*), results in the restriction of infection to the inoculated spikelet because of an enhanced host response (Cuzick *et al*., 2008). In plant cells, DON has been described to both inhibit and induce programmed cell death in a concentration‐dependent manner (Desmond *et al*., 2008; Diamond *et al*., 2013). Hence, in symptomless wheat tissues, polyamine‐induced DON production by *F. graminearum* may inhibit host defences at the translational level, promoting the establishment of infection throughout the wheat head. Once an advancing hyphal colony has been established, the latter accumulation of DON to a higher titration may then assist in the induction of host cell death, intracellular colonization and the development of visible disease symptoms.

Controlling access to iron is central to the regulation of oxidative stress, in addition to fungal pathogenicity and host immunity in multiple plant and animal pathosystems (Kaplan and Kaplan, 2009; Liu *et al*., 2007). Accordingly, the biosynthetic gene clusters responsible for the production of the extracellular siderophores, TAFC and malonichrome, which scavenge iron from the host (Oide *et al*., 2006), were induced in symptomless wheat tissue. In *F. graminearum*, the loss of the ability to secrete TAFC or malonichrome results in reduced virulence and in the case of TAFC, the wheat plant subsequently mounts an enhanced defensive response (Oide *et al*., 2006). Extracellular siderophore‐mediated iron acquisition may influence the pathogen's capacity to withstand the general impact of nutrient, hypoxic and oxidative stresses. More specifically, hyphal exposure to a plant defence‐activated oxidative burst, which often represents the plant's immediate response to the detection of a fungal invader (Lamb and Dixon, 1997), may be reduced in the presence of secreted siderophores. The reductive iron assimilation pathway, which can sustain life in the absence of siderophores, is insufficient to permit pathogenicity (Oide *et al*., 2015), again demonstrating the importance of TAFC and malonichrome to the establishment of infection. A fungal siderophore from *Colletotrichum graminicola* also acts as a pathogen‐associated molecular pattern, activating plant immunity (Albarouki *et al*., 2014). Therefore, both DON and TAFC may minimize the impact of host defences via distinct mechanisms, whilst also having the potential to induce host responses. Interestingly, DON and TAFC biosynthesis originates from shared precursors, such as ornithine from the polyamine pathway and mevalonate from the isoprenoid pathway (Gardiner *et al*., 2010; Haas, 2012), whereas the isoprenoid pathway is regulated by the *Tri6* transcription factor (Nasmith *et al*., 2011). Therefore, both the secreted metabolites DON and TAFC appear to be required for the establishment of symptomless infection and the expansion of infection throughout the wheat head. The role of the many other characterized and putative secondary metabolites remains to be determined.

The *F. graminearum* genome is predicted to code for 574 secreted proteins that are hypothesized to be involved in pathogenicity (Brown and Hammond‐Kosack, 2015; Brown *et al*., 2012). This predicted secretome includes 60 small, predominantly cysteine‐rich, candidate effectors, as predicted by EffectorP (Sperschneider *et al*., 2016), reminiscent of candidate effectors in numerous biotrophic and hemibiotrophic fungal pathogens (Brown and Hammond‐Kosack, 2015; Brown *et al*., 2012). Many of these candidate *F. graminearum* effectors were induced during wheat infections, including 18 with elevated transcript abundance in symptomless wheat tissue, and 22 with elevated transcripts in symptomatic wheat tissue. Hence, putative *Fusarium* effectors with increased abundance in symptomless tissue may potentially promote symptomless infection, whereas putative *Fusarium* effectors with increased transcription in symptomatic tissue may be involved in the induction of host cell death. Therefore, it is highly likely that *F. graminearum* hyphae deploy additional uncharacterized secreted virulence factors to manipulate the host microenvironment and to promote disease formation. Interestingly, the vast majority of genes encoding the aforementioned known and putative proteinaceous or metabolite virulence factors resided within hotspots of high chromosomal recombination, sites previously implicated as regions of diversifying evolution (Cuomo *et al*., 2007), which promote pathogenic interactions and adaptations to live on and in different host plant species.

Similar to classical necrotrophic fungi, such as *Botrytis cinerea* and *Sclerotinia sclerotiorum*, the *F. graminearum* genome is predicted to code for 109 CAZymes involved in plant cell wall degradation (Brown and Hammond‐Kosack, 2015; Brown *et al*., 2012; Heard *et al*., 2015). Interestingly, 50 CAZymes were induced throughout infection, but to a higher level in symptomatic wheat tissue. Possibly, the initial basal secretion of CAZymes in symptomless wheat tissue could facilitate the passage of fungal hyphae within the intercellular space, i.e. between living wheat cells (Brown *et al*., 2010). Increased CAZyme expression correlated with the later phase of symptomatic *F. graminearum* infection, which is represented by the hyphal penetration of dead host cells, the enzymatic degradation of plant cell walls, the appearance of ghost hyphae devoid of content and the macroscopic appearance of bleached wheat tissues (Brown *et al*., 2010, 2011). In fungi, CAZyme activity and the autophagy process are well known to be repressed by freely available sugars (Brown *et al*., 2014). The initial induction of CAZymes by *F. graminearum* in symptomless wheat tissues suggests that sugars are not freely available. This is reminiscent of the limited induction of scavenging CAZymes by lignocellulolytic fungi during carbon starvation and during their initial saprophytic colonization of plant biomass (Brown *et al*., 2014). The subsequent increase in CAZyme transcript abundance by *F. graminearum* within symptomatic wheat tissue implies that, at this later phase, the pathogen is exposed to nutrient deprivation, once the integrity of the wheat tissue has been compromised, and that CAZyme‐inducing saccharides, such as cellobiose and xylobiose (Brown *et al*., 2014), have been released from the symptomatic wheat tissues, resulting in a higher transcriptional induction of fungal CAZymes. Despite these two processes occurring during the later phase of infection, CAZymes, including CbhC1, Eng1 and XylA (FGSG_3624) (Sella *et al*., 2013; Sperschneider *et al*., 2016; Zhang *et al*., 2012), and the autophagy process (Josefsen *et al*., 2012), have been shown previously to be required for disease formation in wheat.

In addition to the arsenal of toxic or non‐toxic metabolite and proteinaceous compounds, several genes encoding for proteins involved in intracellular processes associated with fungal virulence also showed increased transcript abundance in symptomless wheat tissue. These included the previously described pathogenicity‐associated genes identified in pathogenic but not free‐living fungal species, which also show hallmarks of being exposed to diversifying selection (Sperschneider *et al*., 2015). In addition, several PHI‐base genes have been experimentally proven to be involved in *F. graminearum* infection, where their genetic disruption/deletion resulted in a loss of pathogenicity, reduced virulence or hypervirulence (Brown *et al*., 2016; Urban *et al*., 2015). These results again highlight the utility of combining resources and datasets to identify genes important to the pathogenic process in one or more host species.

In conclusion, using an *in planta* transcriptomics approach to explore the *F. graminearum* transcriptome, it has been possible to spatially separate the symptomless and symptomatic aspects of the wheat head infection process. This study has also helped to further define the subsets of *F. graminearum* genes specifically expressed in a single cereal host species (namely wheat, barley or maize) and to define expression combinations evident across two or more cereal hosts. The new FHB infection model for wheat arising from this study is presented in Fig. 7. This model provides insights into the coordination of the multifaceted virulence strategy deployed by the advancing *F. graminearum* hyphal network to establish and then simultaneously maintain both symptomless and symptomatic wheat infection. The placement of fungal genes required for virulence in a refined spatial temporal FHB infection model will help to guide the selection of other *Fusarium* genes for function evaluation via various reverse genetics approaches. The use of this new model should facilitate the discovery of novel *in planta* activated fungal processes and virulence factors required for infection, in turn providing new targets for disease intervention.

## Experimental Procedures

### Fungal propagation, plant cultivation and infections

*Fusarium graminearum* wild‐type strain PH‐1 was cultured, and fresh conidia were prepared as described previously (Brown *et al*., 2010). The susceptible spring wheat (*Triticum aestivum*) cultivar, Bobwhite, was grown and infected as described previously (Brown *et al*., 2010, 2011). In total, 15 inoculated heads were used per treatment per experiment, and the experiment was repeated three times. The macroscopic appearance of disease on representative plants was photographed on a Nikon D40X (Nikon, UK) under natural light, and the sequential rachis internodes below the inoculated spikelet were imaged on a Leica MZFL11 stereomicroscope (Leica Microsystems, UK) under bright field light.

### RNA extraction and RT‐PCR analysis

The seven rachis internodes below the inoculated spikelet, plus the inoculated spikelets of 15 wheat heads, were excised and frozen in liquid nitrogen. Freeze‐dried samples were ground in liquid nitrogen with a pestle and mortar. Total RNA was extracted as described previously (Brown *et al*., 2011). To detect fungal infection, cDNA was synthesized from 1 µg of RNA using 500 ng of OligodT primers and 200 U Superscript III (Invitrogen, UK) according to the manufacturer's instructions. The presence of fungal RNA was confirmed by RT‐PCR using the fungal γ‐actin primers (5′‐ATGGTGTCACTCACGTTGTCC‐3′ and 5′‐CAGTGGTGGAGAAGGTGTAACC‐3′).

The analysis of gene expression was performed by RT‐qPCR on a Real‐Time PCR System 7500 (Applied Biosystems, UK) using the *TRI4* (5′‐AGACTACTTCAAGGACACTGGCC‐3′ and 5′‐GGTAAGGGAGATTCTCTAGGGTAGC‐3′), *TRI5* (5′‐GATGAGCGGAAGCATTTCC‐3′ and 5′‐CGCTCATCGTCGAATTC‐3′), β‐tubulin (5′‐TCAACATGGTGCCCTTCC‐3′ and 5′‐TTGGGGTCGAACATCTGC‐3′) and γ‐actin primers. A standard curve of gDNA with known concentrations was created for each primer pair. Synthesized cDNA was diluted 1 : 50 in sterile H_2_O and PCR was performed using Jumpstart Taq Ready Mix plus ROX (Invitrogen, UK). The average cycle threshold value for each sample was calculated from triplicate technical replicates and the analysis of three biological replicates. The fungal housekeeping genes γ‐actin and β‐tubulin were used to normalize the RT‐qPCR data for fungal biomass.

### Affymetrix hybridization

The RNAs from pairs of rachis internodes with the same macroscopic symptoms were combined from 15 wheat heads, including symptomless infection (RI5 + RI6), intermediate disease symptoms (RI3 + RI4) and fully symptomatic infection (RI1 + RI2). The RNA from the inoculated spikelet (SP1) was not combined. Three independent biological replicates for each tissue per treatment at 7 dpi were analysed. Purified total RNA (>10 µg) was *in vitro* transcribed, labelled using the BioArray high‐yield RNA transcript labelling kit (T7), according to the manufacturer's instructions (Enzo Life Science, UK), fragmented using 200 mm Tris‐acetate (pH 8.2), 500 mm MgOAc, and mixed with the BioArray Eukaryotic hydridization controls (Enzo Life Science, UK), which included 3 nm control oligo B2, 20 × control cRNA cocktail, herring sperm (10 mg/mL), acetylated bovine serum albumin (BSA) (50 mg/mL), 2 × 2‐(N‐morpholino) ethanesulfonic acid (MES) hybridization buffer and water. A final RNA concentration of 10 µg in 200 µL was hybridized to the *F. graminearum* Affymetrix array (Gueldener *et al*., 2006).

### Bioinformatics and statistical analysis

Affymetrix expression data were RMA and MAS5 normalized and extracted using the Affymetrix Expression Console. The data were deposited at the National Center for Biotechnology Information‐Gene Expression Omnibus (NCBI‐GEO) under the accession number GSE79853. Statistical analyses of the RMA normalized data were performed using the limma package within R statistical software. Linear models were fitted to the data from each probe and moderated *t‐*statistics were computed using an empirical Bayes method. A series of contrasts was then applied to the resulting models to answer specific questions. The first contrast (C1) was used to identify the probes which were up‐regulated during infection in comparison with *in vitro* growth in complete medium (Gueldener *et al*., 2006). In addition, three *in planta* contrasts were used: a rachis versus spikelet comparison (C2), a symptomless versus symptomatic rachis tissue comparison (C3) and a comparison of the intermediate rachis tissue with the mean of the combined symptomless and symptomatic rachis tissues (C4). Significance was determined by application of a Benjamini Hochberg multiple testing correction and a significance level of 0.05. Genes which were not significantly up‐regulated during wheat infection or differentially regulated in any of the *in planta* contrasts were then excluded from further consideration before focusing on the exploration of specific results obtained from the *in planta* contrasts. The differentially regulated genes were functionally annotated and mapped onto KEGG metabolic pathways using Blast2GO (Gotz *et al*., 2008). MIPS functional categories (Mewes *et al*., 2004; Ruepp *et al*., 2004) provided additional information via the Pedant interface (http://pedant.helmholtz-muenchen.de/pedant3htmlview/pedant3view?Method=start_method&Db=p3_p13839_Fus_grami_v32). Over‐represented gene ontologies among the differentially regulated genes were identified using Blast2GO (Gotz *et al*., 2008).

Secondary metabolite gene clusters had been predicted previously by Sieber *et al*. (2014). Putative effector proteins within the refined secretome (Brown *et al*., 2012) were predicted using EffectorP (Sperschneider *et al*., 2016). Phenotypic virulence data for all the published *F. graminearum* single‐gene deletion studies were retrieved from PHI‐base version 3.8 (Urban *et al*., 2015). Pathogenicity‐associated genes had been defined previously by Sperschneider *et al*. (2015). The distribution of different gene sets across the four *F. graminearum* chromosomes was displayed using the software OmniMapFree (Antoniw *et al*., 2011).

For comparison, existing *F. graminearum*–wheat Affymetrix datasets were downloaded from www.plexdb.org, including the *in vitro* complete medium, carbon and nitrogen starvation comparison (FG2), the whole wheat head time course experiment (FG15) and the wheat coleoptile LCM study (FG19). Genome‐wide correlations of the RMA normalized data were determined, and hierarchical cluster analysis was performed using complete linkage. The *F. graminearum* genes reported to be preferentially expressed in specific host plants were obtained from Harris *et al*. (2016).

## Supporting information

Additional Supporting Information may be found in the online version of this article at the publisher's website:

**Fig. S1** Fusarium head blight: the macroscopic appearance of wheat disease symptoms throughout the time course of *Fusarium graminearum* infection. (A) The entire wheat head, following point inoculation in the middle two spikelets with *F. graminearum* conidia (isolate PH‐1) or water (mock). (B) The rachis internode assay, utilizing sequential rachis internodes dissected from below the inoculated spikelets at five time points. dpi, days post‐inoculation.

**Fig. S2** Independent validation of the *Fusarium graminearum* Affymetrix expression data via reverse transcription‐quantitative polymerase chain reaction (RT‐qPCR). (A) The expression of γ‐actin and β‐tubulin in symptomless and symptomatic wheat tissues reflects a decrease in fungal biomass. (B) The expression of TRI4 and TRI5 within symptomless and symptomatic wheat tissue. The increased expression of TRI4 and TRI5 in symptomless tissue confirms the findings from the Affymetrix investigation. RI1–RI6, rachis internodes below the inoculated spikelet.

**Fig. S3** The *Fusarium graminearum* genome displayed as four chromosomes providing the locations of gene types with different expression patterns and different recombination frequencies. The following distributions are displayed in the following row order for each chromosome. (A) Chromosomal recombination frequency heatmap (red, high‐level recombination; blue, low‐level recombination) (Cuomo *et al*., 2007). (B) All genes with increased transcript abundance in symptomless wheat rachis tissue. (C) All genes with increased transcript abundance in symptomatic wheat rachis tissue. (D) Genes encoding for secreted proteins with increased transcript abundance in symptomless wheat tissue. (E) Genes encoding all the predicted secondary metabolite clusters. (F) Genes within secondary metabolite clusters with increased transcript abundance in symptomless wheat tissue. (G) Genes coding for predicted transcription factors with increased transcript abundance in symptomless wheat tissue.

**File S1** The differentially regulated *Fusarium graminearum* probes. (1) The 5820 *F. graminearum* probes with increased transcript abundance during *in vitro* culture in complete medium in comparison with during wheat infection. (2) The 4062 *F. graminearum* probes with increased gene transcript abundance during wheat infection compared with *in vitro* culture (C1). (3) The lists of differentially regulated gene transcripts identified by the three *in planta* comparisons: a rachis versus spikelet comparison (C2), a symptomless versus symptomatic rachis tissue comparison (C3) and a comparison of the intermediate rachis tissue with the mean of the combined symptomless and symptomatic rachis tissues (C4).

**File S2** The individual expression values and annotation of the individual transcripts presented in Figures 2–5, including: (1) putative transmembrane transporters; (2) characterized and putative secondary metabolite gene clusters; (3) secreted effector proteins (EffectorP predicted); (4) pathogenicity‐associated; (5) PHI‐base; and (6) secreted hydrolytic enzymes.

**File S3** The 3862 *Fusarium graminearum* genes identified in this study to show increased transcript abundance in different phases of wheat infection compared with those which were previously reported as being specifically or commonly expressed during wheat, barley and maize infection (Harris *et al*., 2016).

**Table S1** The over‐represented gene ontology (GO) terms assigned to the *Fusarium graminearum* genes with increased transcript abundance in wheat rachis tissue compared with spikelet tissue. It should be noted that no over‐represented GO terms were assigned to the genes with increased transcript abundance in the spikelet. BP, biological process; CC, cellular component; MF, molecular function.
**Table S2** A summary of the representation of KEGG (Kyoto Encyclopedia of Genes and Genomes) metabolic pathways among the *Fusarium graminearum* genes differentially modulated during symptomless wheat infection.
