# Supplementary figures and images for: A spatial temporal analysis of the Fusarium graminearum transcriptome during symptomless and symptomatic wheat infection
Source: Mol Plant Pathol. 2017 Aug 8;18(9):1295–312. doi: 10.1111/mpp.12564 (PMC5697668; doi:10.1111/mpp.12564)

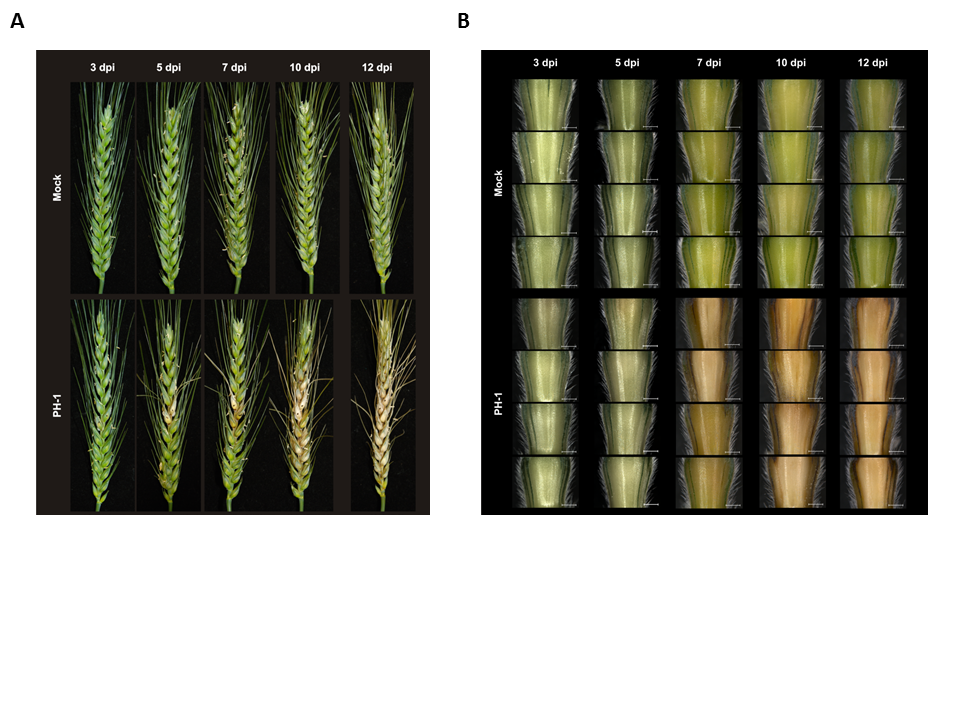

Supplement: Supplementary file 1 — Fig. S1 Fusarium head blight: the macroscopic appearance of wheat disease symptoms throughout the time course of Fusarium graminearum infection. (A) The entire wheat head, following point inoculation in the middle two spikelets with F. graminearum conidia (isolate PH‐1) or water (mock). (B) The rachis internode assay, utilizing sequential rachis internodes dissected from below the inoculated spikelets at five time points. dpi, days post‐inoculation. [file MPP-18-1295-s001.TIF]

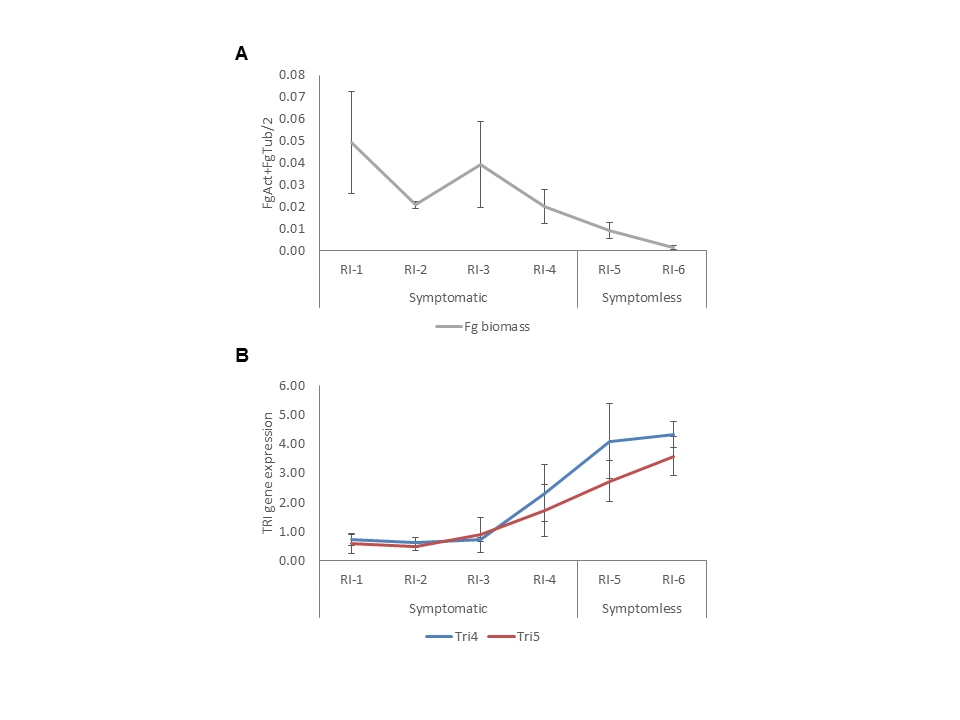

Supplement: Supplementary file 2 — Fig. S2 Independent validation of the Fusarium graminearum Affymetrix expression data via reverse transcription‐quantitative polymerase chain reaction (RT‐qPCR). (A) The expression of γ‐actin and β‐tubulin in symptomless and symptomatic wheat tissues reflects a decrease in fungal biomass. (B) The expression of TRI4 and TRI5 within symptomless and symptomatic wheat tissue. The increased expression of TRI4 and TRI5 in symptomless tissue confirms the findings from the Affymetrix investigation. RI1–RI6, rachis internodes below the inoculated spikelet. [file MPP-18-1295-s002.TIF]

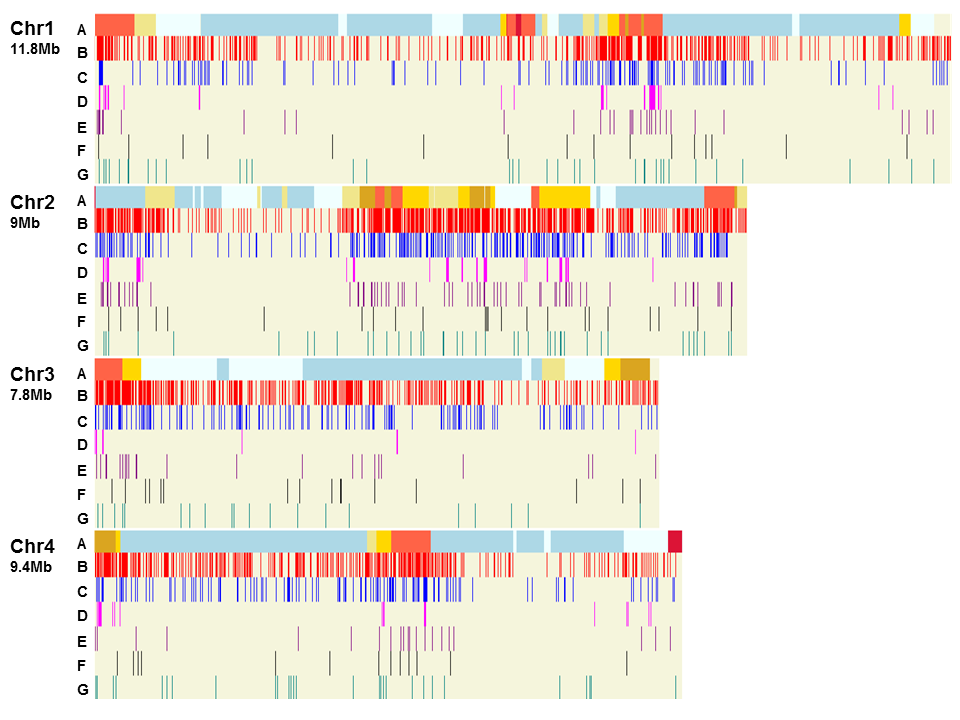

Supplement: Supplementary file 3 — Fig. S3 The Fusarium graminearum genome displayed as four chromosomes providing the locations of gene types with different expression patterns and different recombination frequencies. The following distributions are displayed in the following row order for each chromosome. (A) Chromosomal recombination frequency heatmap (red, high‐level recombination; blue, low‐level recombination) (Cuomo et al., 2007). (B) All genes with increased transcript abundance in symptomless wheat rachis tissue. (C) All genes with increased transcript abundance in symptomatic wheat rachis tissue. (D) Genes encoding for secreted proteins with increased transcript abundance in symptomless wheat tissue. (E) Genes encoding all the predicted secondary metabolite clusters. (F) Genes within secondary metabolite clusters with increased transcript abundance in symptomless wheat tissue. (G) Genes coding for predicted transcription factors with increased transcript abundance in symptomless wheat tissue. [file MPP-18-1295-s003.TIF]
