# Supplementary material for: A spatial temporal analysis of the Fusarium graminearum transcriptome during symptomless and symptomatic wheat infection
Source: Mol Plant Pathol. 2017 Aug 8;18(9):1295–312. doi: 10.1111/mpp.12564 (PMC5697668; doi:10.1111/mpp.12564)
Supplement: Supplementary file 7 — Table S1 The over‐represented gene ontology (GO) terms assigned to the Fusarium graminearum genes with increased transcript abundance in wheat rachis tissue compared with spikelet tissue. It should be noted that no over‐represented GO terms were assigned to the genes with increased transcript abundance in the spikelet. BP, biological process; CC, cellular component; MF, molecular function. Table S2 A summary of the representation of KEGG (Kyoto Encyclopedia of Genes and Genomes) metabolic pathways among the Fusarium graminearum genes differentially modulated during symptomless wheat infection. [file MPP-18-1295-s007.docx]

**Supplementary Table S1** The overrepresented GO terms assigned to the *F. graminearum* genes with increased transcript abundance in wheat rachis tissue compared to spikelet tissue. Note - no overrepresented GO terms were assigned to the genes with increased transcript abundance in the spikelet. BP = biological process, CC = cellular component, MF = molecular function.

| GO-ID | Term | Category | FDR | P-Value | Genes |
| --- | --- | --- | --- | --- | --- |
| 0055085 | Transmembrane transport | BP | 6.08E-05 | 1.26E-08 | 124 |
| 0055114 | Oxidation-reduction process | BP | 3.69E-03 | 2.29E-06 | 165 |
| 0004497 | Monooxygenase activity | MF | 7.52E-03 | 6.82E-06 | 40 |
| 0016021 | Integral component of membrane | CC | 7.52E-03 | 7.76E-06 | 146 |
| 0030247 | Polysaccharide binding | MF | 2.88E-02 | 5.35E-05 | 9 |

**Supplementary Table S2** A summary of the representation of KEGG metabolic pathways among the *Fusarium graminearum* genes differentially modulated during symptomless wheat infection

| **KEGG pathways** | **Up-regulated**  **genes** | **Down-regulated**  **genes** |
| --- | --- | --- |
| Biosynthesis of antibiotics | 11 | 8 |
| Purine metabolism | 8 | 9 |
| Amino sugar and nucleotide sugar metabolism | 7 | 9 |
| Glycine, serine and threonine metabolism | 6 | 0 |
| Pantothenate and CoA biosynthesis | 6 | 0 |
| Aminoacyl-tRNA biosynthesis | 5 | 0 |
| Pentose and glucuronate interconversions | 5 | 5 |
| Oxidative phosphorylation | 5 | 0 |
| Starch and sucrose metabolism | 5 | 10 |
| Pyrimidine metabolism | 5 | 0 |
| Tryptophan metabolism | 0 | 4 |
| Other glycan degradation | 0 | 4 |
| Glycosphingolipid biosynthesis - ganglio series | 0 | 3 |
| Sphingolipid metabolism | 0 | 3 |
| Glyoxylate and dicarboxylate metabolism | 0 | 3 |
